# Factors Associated with Survival to Hospital Discharge in Cardiac Arrest by Poisoning: WAIVOR Score

**DOI:** 10.5811/westjem.47064

**Published:** 2025-11-18

**Authors:** Min-Su Cha, Myoung-Je Song, Jong-Sun Kim

**Affiliations:** International St. Mary’s Hospital, Catholic Kwandong University College of Medicine, Department of Emergency Medicine, Incheon Metropolitan City, Korea

## Abstract

**Introduction:**

Poisoning-induced out-of-hospital cardiac arrest (P-OHCA) is a leading mortality cause; however, no specific prognostic model exists for P-OHCA. In this study we aimed to develop and validate a novel scoring system, the WAIVOR score, which identifies factors associated with survival to hospital discharge in patients with P-OHCA, including the nature of the toxic agent.

**Methods:**

In this retrospective nationwide observational study we analyzed 4,252 South Korean adult P-OHCA cases from 2013–2023. The study population was randomly stratified into derivation (n = 2,834) and validation (n = 1,418) cohorts. Independent factors associated with survival to hospital discharge were identified through multivariable logistic regression analysis, yielding adjusted odds ratios (aOR) and 95% confidence intervals (CI). We assessed the scoring system’s discriminative performance using the receiver operating characteristic curve and area under the curve (AUC) analysis, with optimal threshold determination via the Youden index.

**Results:**

Among all patients, 291 (6.8%) survived to hospital discharge. The most frequent poisoning substances were gases/vapors (45.3%), pesticides (31.5%), and medically prescribed drugs (12.0%). Six independent factors associated with survival to hospital discharge were incorporated into the WAIVOR score (maximum 11 points): pre-hospital return of spontaneous circulation, four points (aOR 16.11, 95% CI 10.16–25.64); witnessed arrest, two points (aOR 3.86, 95% CI 2.61–5.71); age < 65 years, two points (aOR 3.34, 95% CI 2.20–5.15); female sex, one point (aOR 1.54, 95% CI 1.09–2.16); and arrest-to-emergency department intervals ≤ 30 minutes, two points (aOR 3.44, 95% CI 2.00–6.09; 31–60 minutes, one point (aOR 1.77, 95% CI 1.08–3.02); and poisoning by non-gas/non-vapor substances, one point (aOR 0.54, 95% CI 0.33–0.89). The WAIVOR score demonstrated robust discriminative performance (AUC: 0.823 and 0.739 in derivation and validation cohorts, respectively). At the optimal threshold of five points, the score demonstrated 53.6% sensitivity, 84.4% specificity, 19.8% positive predictive value, and 96.2% negative predictive value (NPV).

**Conclusion:**

The WAIVOR score represents a practical tool whose associated factors may help assess potential for survival to hospital discharge in patients with P-OHCA. Its high NPV renders it valuable for identifying poor prognostic outcomes. However, further external validation studies are required before this score can be broadly used in decisions regarding resuscitation termination in clinical practice.

## INTRODUCTION

Out-of-hospital cardiac arrest (OHCA) remains a leading cause of global mortality, associated with poor overall prognosis and neurological outcomes.^1^ The etiology of OHCA can be categorized into medical and non-medical causes, including trauma, drowning, asphyxia, and poisoning.^2^ In 2023, 105,007 drug overdose deaths occurred in the United States, resulting in an age-adjusted rate of 31.3 deaths per 100,000 standard population, of which opioid overdoses accounted for approximately 24.0 deaths per 100,000.^3^ Poisoning-induced OHCA (P-OHCA) accounts for approximately 6% of non-traumatic OHCAs, resulting in substantial societal and economic burdens.^4^

Patients with P-OHCA are younger, experience fewer witnessed events, and present with lower rates of shockable rhythms than do those with non-poisoning OHCA.^5^ The clinical outcomes for patients with P-OHCA vary across studies; however, recent studies have reported generally better outcomes than those of patients with non-poisoning OHCA, with survival rates with good neurological outcome of 15.2% vs 8.8% (P < .001), and an adjusted odds ratio (aOR) of 2.47.^6^,^7^ Given the reported favorable clinical outcomes of P-OHCA compared to other OHCA etiologies, elucidating its specific prognostic factors is essential for optimizing patient outcomes.

Various prediction models have been developed to assess clinical outcomes in OHCA.^8^ However, the unique pathophysiology and diverse toxic agents involved in P-OHCA make it challenging to accurately predict outcomes using existing prediction models. This underscores the need for a prognostic model tailored to P-OHCA cases, which can serve as an essential clinical tool for improving resource allocation and patient outcomes.

Considering the low prevalence but generally favorable clinical outcomes in patients with P-OHCA, a risk-stratification system specific to P-OHCA for predicting clinical outcomes is critical. However, previous tools have not fully considered the distinct characteristics of patients with P-OHCA. Therefore, our goal in this study was to develop a novel scoring system for identifying survival to hospital discharge specific to patients with P-OHCA.

## METHODS

### Study Design and Data Collection

In this retrospective observational study we used the Out-of-Hospital Cardiac Arrest Surveillance (OHCAS) database maintained by the Korea Disease Control and Prevention Agency (KDCA). The OHCAS is a nationwide registry and represents the largest and most comprehensive source of standardized data on OHCA in South Korea, annually capturing detailed information on approximately 30,000 patients with cardiac arrest who receive emergency medical service (EMS) care and subsequent transportation to medical facilities.

The OHCAS database integrates information from the National Fire Agency database and hospital medical records validated by the KDCA. The initial dataset originates from EMS documentation, encompassing clinical information, such as cardiac arrest circumstances, resuscitation timing, and prehospital interventions. This information is systematically recorded by EMS responders during prehospital care and transport. The second component involves a comprehensive hospital medical record review. Trained KDCA investigators conduct on-site visits to healthcare facilities to collect detailed information about in-hospital care, interventions, toxic exposures, and patient outcomes. The review process follows standardized protocols aligned with the Utstein-style guidelines and Resuscitation Outcomes Consortium Project parameters. Additionally, this retrospective chart review complied with recommended methodological criteria by Worster et al (2005), including clearly defined inclusion and exclusion criteria, abstractor training prior to data collection, explicit variable definitions, clear identification of the medical record databases, systematic assessment of interobserver reliability, defined management plans for missing data, and obtaining institutional review board approval.^9^

**Population Health Research Capsule**

What do we already know about this issue?

*Poisoning-induced out-of-hospital cardiac arrest (P-OHCA) has unique prognostic factors but lacks a risk-stratification system for predicting clinical outcomes.*

What was the research question?

*Can we develop and internally validate a risk-stratification model for P-OHCA?*

What was the major finding of the study?

*The WAIVOR score demonstrated an area under the curve of 0.739 and a negative predictive value of 96.2%.*

How does this improve population health?

*The WAIVOR score is a valuable tool for guiding clinical decision-making and optimizing resource allocation in P-OHCA, including decisions on termination or prolonged resuscitation.*

### Study Setting and Emergency Medical Services in South Korea

This study was conducted within the context of South Korea’s national EMS infrastructure. The EMS system operates on a 24/7 basis under a government-operated model. Upon identification of OHCA, EMS personnel initiate an immediate response following standardized protocols. Under medical direction, EMS responders are authorized to perform clinical interventions, including advanced airway management and the administration of intravenous (IV) fluids (isotonic or dextrose), supplementary oxygen, bronchodilator nebulizer, sublingual nitroglycerin, and IV or intramuscular epinephrine under specific clinical situations. However, administration of antidote medications for poisoning or envenomation (eg, naloxone, flumazenil, glucagon, ethanol, N-acetylcysteine, atropine, pralidoxime, and anti-venom) and gastric decontamination interventions are not permitted in the prehospital phase.

The EMS responders transport all patients with OHCA to the nearest emergency department (ED). As per South Korean EMS protocols, field pronouncement of death by EMS personnel is not permitted. Consequently, all patients with EMS-attended OHCA are transported to healthcare facilities and subsequently included in the OHCAS database. However, in cases where resuscitation is deemed futile due to obvious signs of death (including rigor mortis, livor mortis, or decapitation), EMS responders may withhold resuscitative efforts following online medical consultation. These cases are subsequently managed via private ambulance transport and are excluded from the OHCAS database.

### Study Participant Selection

In the OHCAS database (2013–2023), we excluded cases in which the cause of OHCA was not poisoning, pediatric patients (< 19 years of age), patients with do-not-resuscitate (DNR) orders, and those with incomplete clinical outcome data. Given the large-scale clinical data and the rarity of survival to hospital discharge in P-OHCA, we performed internal validation using a split-sample method.^10^ Accordingly, the study population was randomly stratified into a derivation cohort, comprising two-thirds of it, and a validation cohort, comprising the remaining one-third.

### Variables and Measurements

We examined various variables encompassing patient demographics, cardiac arrest circumstances, and emergency response metrics. Demographic variables included age, sex, and residence (metropolitan vs non-metropolitan/rural). Cardiac arrest characteristics were systematically documented through the following parameters: location of arrest (public vs non-public); witnessed cardiac arrest; bystander cardiopulmonary resuscitation (CPR); first monitored rhythm (shockable vs non-shockable); and achievement of prehospital return of spontaneous circulation (ROSC). The arrest-to-ED interval time was defined as the temporal duration from cardiac arrest onset to ED arrival. Determination of arrest time was based on direct witness accounts or information obtained from emergency callers or caregivers. In cases where precise arrest timing could not be established, the last documented time of normal health status was designated as the reference point. To enhance scoring system simplicity, we transformed continuous variables into categorical variables: age was dichotomized at 65 years; and the arrest-to-ED interval was stratified using 30- and 60-minute thresholds.

Cardiac arrest etiologies in the OHCAS database are classified into predefined categories, including “poisoning.” We included only cases coded as “poisoning” to ensure consistent exclusion of non-poisoning etiologies. As per the OHCAS data collection protocol, not all ingested or exposed agents are routinely recorded. While it is possible to document multiple poisoning substances, trained investigators systematically review both EMS records and hospital medical charts to identify and record the substances responsible for triggering the cardiac arrest. Based on a previously described classification method, poisoning substances were categorized into five groups:^5^,^11^ medically prescribed drugs, including non-opioid analgesics, antipyretics, antirheumatics, antiepileptic sedative-hypnotics, and other unspecified medicinal and biological substances; gases and vapors; pesticides, including insecticides and herbicides; alcohol-based substances, including ethanol, methanol, organic solvents, and halogenated hydrocarbons; and unspecified chemicals and biological toxins. A detailed list of poisoning substances belonging to each group is provided in Appendix 1.

### Outcome Measurement

The primary endpoint was survival to hospital discharge, defined as either discharge to home or transfer to another healthcare facility following completion of acute medical management. We ascertained discharge status through a comprehensive review of hospital discharge documentation, which was initially documented by the attending physicians.

### Statistical Analysis

We performed statistical analyses using R software v4.4.2 (The R Foundation for Statistical Computing, Vienna, Austria). Descriptive statistics are presented as medians with interquartile ranges for continuous variables and as numbers with percentages for categorical variables. Baseline characteristics were compared using the Mann–Whitney U test for non-parametric continuous variables and the chi-square test for categorical variables. We identified predictors of survival to hospital discharge through multivariable logistic regression analysis, with adjustment for potential confounders identified in univariate analysis. The regression model generated adjusted odds ratios (aOR) with corresponding 95% confidence intervals (CIs). Based on these regression coefficients, we developed a clinical prediction scoring system. The scoring system’s discriminative performance was assessed using receiver operating characteristic (ROC) curve analysis with the calculation of the area under the curve (AUC). We determined the optimal cutoff value for the scoring system using the Youden index. Statistical significance was set at *P* < .05.

## RESULTS

### Study Participants

**Figure 1:**
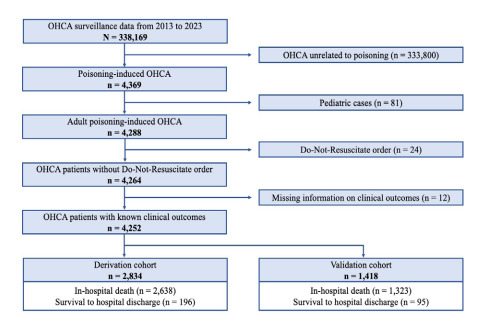
Flowchart illustrating participant selection process for the development and validation of the WAIVOR score. *OHCA*, out-of-hospital cardiac arrest.

Between 2013–2023, 338,169 OHCA cases were registered in the OHCAS database. We first excluded 333,800 cases that were unrelated to poisoning. In the remaining poisoning-related cases, we excluded 81 pediatric cases (< 19 years of age), 24 cases with DNR orders, and 12 cases with incomplete clinical outcome data. The final study cohort comprised 4,252 eligible participants. The study population was randomly stratified into a derivation cohort (n = 2,834) and a validation cohort (n = 1,418) (Figure 1).

The median age of the total study population (n = 4,252) was 56 years, with 64.1% of patients younger than 65 years of age. Male patients accounted for 65.7% of the overall cohort. Most cases occurred in non-metropolitan and rural areas (70.7%), and cardiac arrests predominantly took place in non-public locations (87.0%). Overall, 15.4% of arrests were witnessed, bystander response occurred in 23.4%, shockable initial rhythms were documented in 1.5%, and prehospital ROSC was achieved in 3.8%. The most frequently identified poisoning substances were gases and vapors (45.3%), followed by pesticides (31.5%). The median arrest-to-ED interval was 46 minutes, with most cases (57.0%) presenting within 31–60 minutes. Survival to hospital discharge was observed in 6.8% of the total cohort. All demographic and clinical characteristics were comparable between the derivation and validation cohorts. Detailed baseline characteristics of the total population, derivation cohort (n = 2,834), and validation cohort (n = 1,418) are presented in Table 1.

### Predictors of Survival to Hospital Discharge

Table 2 summarizes the univariate and multivariable analyses of factors associated with survival to hospital discharge in the derivation cohort. Univariate analysis revealed age < 65 years of age (77.0% vs 63.3%, *P* < .001), female sex (43.8% vs 34.1%, *P* < .01), witness (50.0% vs 12.9%, *P* < .001), bystander response (39.7% vs 22.6%, *P* < .001), shockable rhythm (6.6% vs 1.0%, *P* < .001), and achievement of prehospital ROSC (29.5% vs 2.1%, *P* < .001) to be significantly associated with survival to hospital discharge. Additionally, both the type of poisoning agent and the arrest-to-ED interval time demonstrated significant associations with survival to discharge (both *P* < .001).

Multivariable logistic regression analysis identified six independent predictors of survival to hospital discharge. Achievement of prehospital ROSC demonstrated the strongest association (aOR 16.11, 95% CI 10.16–25.64, *P* < .001), followed by witnessed cardiac arrest (aOR 3.86, 95% CI 2.61–5.71, *P* < .001) and age < 65 years (aOR 3.34, 95% CI 2.20–5.15, *P* < .001). Female sex maintained statistical significance (aOR 1.54, 95% CI 1.09–2.16, *P* = .01). Regarding time intervals, shorter arrest-to-ED interval was associated with improved survival: interval time ≤ 30 minutes (aOR 3.44, 95% CI 2.00–6.09, *P* < .001) and 31–60 minutes (aOR 1.77, 95% CI 1.08–3.02, *P* = .02) demonstrated better outcomes than did interval time ≥ 61 minutes. Among poisoning substances, only gases and vapors exhibited significantly reduced survival probability compared to medically prescribed drugs (aOR 0.54, 95% CI 0.33–0.89, *P* = .01).

### Development of the WAIVOR Score

**Figure 2:**
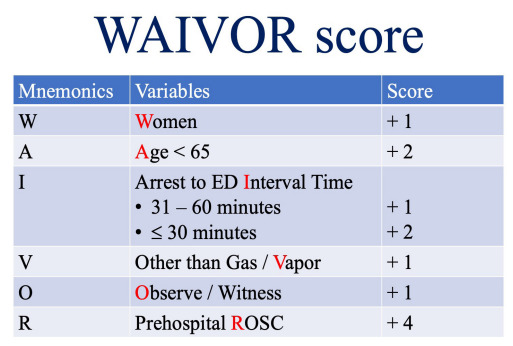
Components of the WAIVOR score, including weighted points assigned based on multivariable logistic regression analysis. *ED*, emergency department; *ROSC*, return of spontaneous circulation.

Based on the multivariable analysis results, we developed a novel scoring system for predicting survival to hospital discharge in P-OHCA. The scoring system incorporates six independent predictors that demonstrated significant associations with survival to hospital discharge in the multivariable model: women, young age (< 65 years), arrest-to-ED interval time, poisoning substance (other than gas or vapor), witness, and prehospital ROSC. The weights for each variable were derived from the regression coefficients in the multivariable logistic regression model, yielding a maximum possible score of 11 points (Figure 2).

### Predictive Performance of the WAIVOR Score

**Figure 3:**
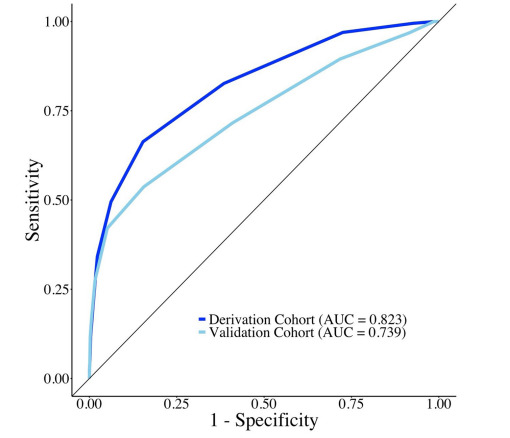
Receiver operating characteristic curves demonstrating the predictive performance of the WAIVOR score for survival to hospital discharge among patients with poisoning-induced out-of-hospital cardiac arrest in both derivation and validation cohorts. *AUC*, area under the receiver operating characteristic curve.

The WAIVOR score demonstrated favorable predictive accuracy with AUCs of 0.823 and 0.739 in the derivation and validation cohorts, respectively (Figure 3). Using the Youden index, we identified a WAIVOR score threshold of five points as the optimal cutoff for predicting survival to hospital discharge, yielding a sensitivity of 66.3% and specificity of 84.6% in the derivation cohort.

Regarding clinical outcomes, survival to hospital discharge was observed in 291 patients (6.8%) of the total cohort (N = 4,252). Specifically, 196 (6.9%) of the derivation cohort (n = 2,834) and 95 (6.6%) of the validation cohort (n = 1,418) survived to hospital discharge. Application of the optimal cutoff (WAIVOR score of 5) in the validation cohort demonstrated a sensitivity of 53.6% and specificity of 84.4%. Within the validation cohort, patients with WAIVOR scores ≤ 4 constituted 66.2% of in-hospital deaths and 2.1% of survivors, whereas those with scores ≥ 5 represented 27.0% of in-hospital deaths and 4.5% of survivors (Table 3). These findings corresponded to a positive predictive value (PPV) of 19.8% and a negative predictive value (NPV) of 96.2%.

## DISCUSSION

To our knowledge, this study presents the first clinical outcome prediction model specifically developed for P-OHCA. The WAIVOR score incorporates six independent predictors: women; age < 65 years; arrest-to-ED interval time; poisoning substance (other than gas or vapor); witnessed cardiac event; and prehospital ROSC. The scoring system demonstrated good predictive performance, with a maximum attainable score of 11 points and an optimal cutoff of five points. The model’s utilization of readily available clinical parameters will facilitate rapid implementation in emergency settings.

The WAIVOR score demonstrated a notably high NPV of 96.2%, indicating its potential utility in identifying patients with P-OHCA with unfavorable prognostic outcomes (in-hospital death) and making decisions regarding the continuation of resuscitation efforts. Although the universal termination of resuscitation (TOR) guideline has demonstrated excellent predictive performance, it possesses significant limitations for P-OHCA cases.^12^,^13^ The universal TOR guideline was primarily validated in cardiac-origin arrests, excluding non-cardiac etiologies; it was designed for pre-transport decision-making by EMS personnel, limiting its applicability to ED-based decisions in P-OHCA cases. Subsequent studies have developed and validated ED-based TOR criteria in the Japanese population, achieving high PPV for unfavorable neurological outcomes and one-month mortality.^14^,^15^ However, these studies predominantly comprised cardiac-origin arrests (48.2–55.7% of study populations), potentially limiting their generalizability to P-OHCA cases.

Emergency physicians currently face significant challenges due to the absence of objective, evidence-based prognostic tools specifically tailored for patients with P-OHCA. The primary contribution of our study lies in addressing this critical clinical gap by proposing the first systematic approach to stratifying prognosis in this unique patient population. While we emphasize that clinical decisions regarding TOR should not rely solely on the WAIVOR score, this tool offers an initial evidence-based framework to support structured prognostic discussions and informed decision-making. Ultimately, our study serves as a foundational step toward establishing more robust, evidence-driven prognostication strategies for P-OHCA and is intended to encourage further external validation and clinical application research in this challenging domain.

According to the current guidelines of the European Resuscitation Council, clinicians are encouraged to prepare for prolonged resuscitation efforts and consider the use of extracorporeal cardiopulmonary resuscitation (ECPR) in cases of cardiac arrest due to poisoning.^16^ In this context, the WAIVOR score, particularly when below the defined threshold, may serve as a helpful tool to support clinical decision-making regarding the appropriateness of conventional vs prolonged resuscitation strategies or the potential candidacy for ECPR. However, further prospective research is needed to validate its utility in such applications.

The relatively low PPV of 19.8% at the optimal cutoff of five points can be attributed to the low baseline prevalence of survival to hospital discharge in P-OHCA cases. In low-prevalence conditions, such as survival in P-OHCA, PPV is inherently low, whereas NPV remains high, which is a known statistical phenomenon.^17^ Given the prevalence-dependent nature of predictive values, the low frequency of survival outcomes inherently constrains the proportion of true positives among predicted positives. These findings underscore the importance of interpreting PPV within the context of low-prevalence conditions and emphasize the clinical utility of the WAIVOR score in identifying poor prognostic outcomes, as evidenced by its high NPV.

The AUC demonstrated excellent discriminative performance in the derivation cohort (AUC = 0.823) and good performance in the validation cohort (AUC = 0.739). The AUC observed in the derivation cohort may be inflated due to inherent overfitting of the prediction model to the derivation dataset.^18^ Additionally, the smaller size of the validation cohort may have increased statistical variability in AUC estimation.

Although numerous prognostic scoring systems have been developed for OHCA, their direct comparison with the WAIVOR score was precluded in this study for the following reasons. First, existing prediction models have been developed largely for patients with OHCA and cardiac or medical etiologies, which differ from the patient population in this study. Second, various prediction models target different clinical outcomes. A recent review identified 16 prediction models for OHCA, with 10 models predicting different clinical outcomes (ROSC, neurological outcomes, and long-term outcomes).^8^ Third, the OHCAS database lacks several variables used in other prediction models. The prognostic scores predicting survival to hospital discharge (NULL-PLEASE score, PCAC score, CREST score, PEA score, and GCS) require specific clinical parameters: laboratory (serum pH and lactate); imaging (cardiac ejection fraction); and physical examination (neurological motor and brainstem responses) results.^8^ These parameters are unavailable in the OHCAS dataset. However, such detailed clinical information is often unavailable during the initial ED presentation of patients with cardiac arrest. The WAIVOR score uses readily available parameters derived from EMS personnel or patient caregivers. Although future external validation studies comparing the WAIVOR score with existing prediction models are warranted, its simplicity and real-time applicability may confer superior clinical utility in emergency settings, even if other prediction models exhibit higher predictive performance.

Regarding poisoning substances, P-OHCA caused by gases and vapors demonstrated significantly lower survival to discharge compared to P-OHCA caused by medically prescribed drugs, aligning with two previous studies.^5^,^11^ However, while two prior studies reported significantly reduced survival in pesticide-related P-OHCA, our study did not demonstrate such an association. This discrepancy may be attributed to temporal changes, as our study encompassed more recent data (2013–2023). Pesticide self-poisoning has shown a global declining trend.^19^ In South Korea, specifically, regulatory interventions have substantially altered P-OHCA patterns. The 2012 paraquat ban, implemented in response to its frequent use in self-poisoning, has contributed to annual reductions in pesticide ingestion cases and a 10% decrease in national suicide rates.^20^–^22^ Consequently, the use of more contemporary data in our study may explain the attenuated impact of pesticides on survival to hospital discharge.

In this study, the survival to hospital discharge rate among patients with P-OHCA was 6.8% (n = 291), which is relatively lower than the 9% pooled rate of survival to hospital discharge reported in a prior meta-analysis.^6^ This discrepancy may be explained by differences in the distribution of causative agents. While opioids were the most common etiology in the previous study, the most frequently identified poisoning substances in our cohort were gases and vapors (45.3%), followed by pesticides (31.5%). These two categories together account for 76.8% of cases and are generally associated with high lethality, which may have contributed to the poorer outcomes observed in our study.

Notably, the five remaining variables included in the WAIVOR score, aside from the “gas or vapor” component, are commonly used predictors in previously published general OHCA scoring systems.^8^,^23^ However, unlike cardiac-origin OHCAs, outcomes in P-OHCA are significantly influenced by the nature of the toxic agent. Therefore, even if similar variables are used, the relative prognostic contribution of each factor may vary depending on the underlying etiology of cardiac arrest. In this study, we quantitatively assessed the prognostic impact of each variable specifically within the P-OHCA population using multivariable logistic regression analysis. As a result, we believe that the WAIVOR score offers improved predictive accuracy tailored to P-OHCA. Nonetheless, further prospective multicenter studies are warranted to externally validate these findings.

Our analysis showed that two key predictors, bystander response and shockable rhythm, which are typically associated with favorable outcomes in general OHCA populations, were not independently significant predictors in our P-OHCA cohort. The low prevalence of shockable rhythms and the strong association between prehospital ROSC and clinical outcome may have overshadowed the predictive value of other variables, thereby limiting the statistical power to detect significant associations in the multivariable model.

## LIMITATIONS

This study has some limitations. First, our findings may not be generalizable to pediatric P-OHCA cases or global populations, as our study focused on adult patients in South Korea. Second, the possibility of selection bias cannot be entirely ruled out. Some cases may have been misclassified as P-OHCA despite having non-poisoning etiologies. Additionally, the OHCAS database excludes patients who were not resuscitated due to obvious signs of irreversible death, such as rigor mortis or decapitation. This raises the possibility that acute poisonings involving highly lethal substances with rapid onset, such as cyanide, may have been excluded from our analysis. However, case classification in the OHCAS registry is based on a comprehensive national survey conducted by trained experts, integrating EMS records and hospital data, which likely minimizes the risk of significant misclassification. Third, we did not take into account the quantities of the poisoning agents ingested or exposed to. However, accurate dose estimation is often infeasible in real-world settings due to the unconscious state of patients, absence of reliable witness accounts, and the inherent difficulty in quantifying exposure, particularly for gaseous substances. Although exposure dose could not be incorporated, this was a necessary trade-off to preserve the clinical applicability and practicality of the WAIVOR score in acute care environments. Lastly, this study lacks external validation; however, internal validation was performed to maintain objectivity. Additionally, the OHCAS data represent the largest OHCA database in South Korea, providing a sufficiently representative sample of patients with OHCA.

## CONCLUSION

We developed and validated a simple scoring system, the WAIVOR score, whose elements are associated with the probability of survival to hospital discharge in cases of poisoning-induced out-of-hospital cardiac arrest. This score incorporates key factors, including sex, age < 65 years, arrest-to-ED interval time, poisoning substance (other than gas or vapor), witnessed cardiac arrest, and prehospital ROSC. The WAIVOR score demonstrated robust predictive performance, with high negative predictive value, making it a valuable tool for guiding clinical decision-making and optimizing resource allocation. However, due to the limitations of this study, these findings should be considered preliminary rather than conclusive. Further research is warranted before the WAIVOR score can be established as a guideline and applied in clinical decision-making for patients with P-OHCA. The study’s findings could help establish strategies for enhancing the management and treatment of P-OHCA.

## Supplementary Information



## Figures and Tables

**Table 1:** Baseline demographic and clinical characteristics of patients with poisoning-induced out-of-hospital cardiac arrest.

Variables	Total population (N = 4,252)	Derivation cohort (n = 2,834)	Validation cohort (n = 1,418)	P-value
Age (years)	56 (41–72)	55 (41–72)	56 (41–71)	.78
Young age (≤ 65 years)	2,725 (64.1)	1,822 (64.2)	903 (63.6)	.72
Female sex	1,460 (34.3)	987 (34.8)	473 (33.3)	.35
Residence				.50
Metropolitan	1,245 (29.3)	820 (28.9)	425 (29.9)	
Non-metropolitan/rural	3,007 (70.7)	2,014 (71.0)	993 (70.0)	
Witnessed	656 (15.4)	439 (15.4)	217 (15.3)	.90
Bystander response	995 (23.4)	676 (23.8)	319 (22.4)	.34
Shockable rhythm	62 (1.5)	41 (1.4)	21 (1.4)	1
Arrest in public place	554 (13.0)	378 (13.3)	176 (12.4)	.42
Prehospital ROSC	162 (3.8)	116 (4.0)	46 (3.2)	.20
Poisoning substance				.28
Medically prescribed drugs	509 (12.0)	338 (11.9)	171 (12.0)	
Gases and vapors	1,926 (45.3)	1,315 (46.4)	611 (43.0)	
Pesticides	1,338 (31.5)	876 (30.9)	462 (32.5)	
Alcohol-based substances	82 (1.9)	52 (1.8)	30 (2.1)	
Unspecified and biological toxins	397 (9.3)	253 (8.9)	144 (10.1)	
Multiple substances poisoning	35 (0.8)	26 (0.9)	9 (0.6)	.43
Arrest-to-ED interval time (minutes)	46 (27–105)	46 (27–103)	47 (29–112)	.29
Arrest-to-ED interval time				.21
≥ 61 minutes	1,022 (24.0)	674 (23.7)	348 (24.5)	
31–60 minutes	2,423 (57.0)	1,601 (56.4)	822 (57.9)	
≤ 30 minutes	807 (19.0)	559 (19.7)	248 (17.4)	
Survival to hospital discharge	291 (6.8)	196 (6.9)	95 (6.6)	.84

Continuous variables are presented as medians (interquartile ranges). Categorical variables are presented as numbers (percentages).

*ED*, emergency department; *ROSC*, return of spontaneous circulation.

**Table 2:** Results of univariate and multivariable logistic regression analyses identifying factors associated with survival to hospital discharge among patients with poisoning-induced out-of-hospital cardiac arrest in the derivation cohort.

Variables	Univariate analysis			Multivariable logistic regression analysis		
	
	In-hospital death (n = 2,638)	Survival to discharge (n = 196)	P-value	aOR	95% CI	P-value
Young age (≤ 65 years)	1,671 (63.3)	151 (77.0)	< .001	3.34	2.20–5.15	< .001
Female sex	901 (34.1)	86 (43.8)	< .01	1.54	1.09–2.16	.01
Residence			.34			
Metropolitan	757 (28.6)	63 (32.1)				
Non-metropolitan/rural	1,881 (71.3)	133 (67.8)				
Witnessed cardiac arrest	341 (12.9)	98 (50.0)	< .001	3.86	2.61–5.71	< .001
Bystander response	598 (22.6)	78 (39.7)	< .001	0.83	0.58–1.21	.34
Shockable rhythm	28 (1.0)	13 (6.6)	< .001	1.56	0.63–3.70	.31
Arrest in public place	352 (13.3)	26 (13.2)	1			
Prehospital ROSC	58 (2.1)	58 (29.5)	< .001	16.11	10.16–25.64	< .001
Poisoning substance			< .001			
Medically prescribed drugs	289 (10.9)	49 (25.0)		Reference	-	-
Gases and vapors	1,262 (47.8)	53 (27.0)		0.54	0.33–0.89	.01
Pesticides	816 (30.9)	60 (30.6)		0.79	0.48–1.30	.35
Alcohol-based substances	49 (1.8)	3 (1.5)		0.39	0.06–1.51	.23
Unspecified and biological toxins	222 (8.4)	31 (15.8)		1.21	0.69–2.12	.49
Multiple substances poisoning	23 (0.8)	3 (1.5)	.58			
Arrest-to-ED interval time			< .001			
≥ 61 minutes	650 (24.6)	24 (12.2)		Reference	-	-
31–60 minutes	1,513 (57.3)	88 (44.8)		1.77	1.08–3.02	.02
≤ 30 minutes	475 (18.0)	84 (42.8)		3.44	2.00–6.09	< .001

Continuous variables are presented as medians (interquartile ranges). Categorical variables are presented as numbers (percentages).

*aOR*, adjusted odds ratio; *ED*, emergency department; *ROSC*, return of spontaneous circulation.

**Table 3:** Distribution of clinical outcomes (survival to discharge or in-hospital death) based on WAIVOR score cutoff among patients with poisoning-induced out-of-hospital cardiac arrest in both derivation and validation cohorts.

	Derivation cohort (n = 2,834)		Validation cohort (n = 1,418)	
	
	In-hospital death (n = 2,638)	Survival to discharge (n = 196)	In-hospital death (n = 1,323)	Survival to discharge (n = 95)
WAIVOR score ≤ 4	1,924 (67.8%)	44 (1.5%)	939 (66.2%)	31 (2.1 %)
WAIVOR score ≥ 5	714 (25.1%)	152 (5.3%)	384 (27.0%)	64 (4.5%)

*WAIVOR*, a scoring system to aid in identifying prognostic outcome of survival to hospital discharge in cases of poisoning-related cardiac arrest.
