# Supplementary material for: Factors Associated with Survival to Hospital Discharge in Cardiac Arrest by Poisoning: WAIVOR Score
Source: West J Emerg Med. 2025 Nov 18;26(6):1755–63. doi: 10.5811/westjem.47064 (PMC12698148; doi:10.5811/westjem.47064)
Supplement: Supplementary file 1 [file wjem-26-1755-s001.docx]

**Appendix 1**. Poisoning substances recorded as causes of poisoning-induced out-of-hospital cardiac arrest in the Out-of-Hospital Cardiac Arrest Surveillance data.

| **Medically prescribed drugs** | **Non-opioid analgesics & anti-pyretics**: acetaminophen, aspirin, salicylate, non-steroidal anti-inflammatory drugs (ibuprofen, dexibuprofen, naproxen, diclofenac, ketoprofen, meloxicam, celecoxib, loxoprofen, mefenamic acid), gabapentin, pregabalin  **Anti-rheumatics**: sulfasalazine, azathioprine, cyclosporin, cyclophosphamide, penicillamine  **Anti-epileptics**: topiramate, valproate, carbamazepine, levetiracetam, lamotrigine  **Sedative-hypnotics**: benzodiazepines, barbiturate, propofol, zolpidem, doxylamine  **Anti-Parkinsonism**: levodopa, carbidopa  **Psychotropic drugs**: sertraline, fluoxetine, citalopram, escitalopram, venlafaxine, duloxetine, amitriptyline, nortriptyline, bupropion, haloperidol, chlorpromazine, aripiprazole, olanzapine, quetiapine, risperidone, clozapine  **Opioids**: cocaine, amphetamine, opioid, methadone, fentanyl, cannabis  **Drugs acting on the autonomic nervous system**: epinephrine, norepinephrine, dopamine, terbutaline, mephentermine, beta-blockers |
| --- | --- |
| **Gases and vapors** | Carbon monoxide, formaldehyde, lacrimogenic gas, fluorine, hydrogen fluoride, hydrogen sulfide, methane gas |
| **Pesticides** | Organophosphate, carbamate, pyrethroids, paraquat, glyphosate, glufosinate |
| **Alcohol-based substances** | **Organic solvents and halogenated hydrocarbons**: Paint thinner, volatile organic compound, petroleum products, benzene, glycols, ketones, carbon tetrachloride, chloroform, trichloroethylene, tetrachloroethylene, dichloromethane  **Alcohol**: ethanol, methanol, 2-Propanol, fusel oil^†^, isopropanol |
| **Unspecified and biological toxins** | Tetrodotoxin, jellyfish venom, snake venom, toxic plant |

^†^Fusel oil: a mixture of higher alcohols that are by-products of alcoholic fermentation; also referred to as fusel alcohols or fuselol.
